# Unraveling the Thermal Effects of High-Intensity Ultrasound: A Practical Guide to Acoustic Power Determination and Heat Management

**DOI:** 10.1021/acsomega.4c11498

**Published:** 2025-05-13

**Authors:** Lucas Previtali Ferraz, Eric Keven Silva

**Affiliations:** 28132 Universidade Estadual de Campinas (UNICAMP), Faculdade de Engenharia de Alimentos (FEA), Rua Monteiro Lobato, 80, Campinas, São Paulo CEP 13083-862, Brazil

## Abstract

This study provides a practical guide for determining acoustic power, the actual energy delivered during high-intensity ultrasound (HIUS) processing, which is critical for the effective design of ultrasound-assisted processes. Acoustic energy is fundamental for ensuring precise process scaling and optimization. Additionally, we address a key misconception in the literature, challenging the view that HIUS is a strictly nonthermal treatment. While HIUS has been widely explored for enhancing process efficiency, reducing energy consumption, lowering costs, and minimizing environmental impact in food and beverage processing, its thermal effects have often been overlooked. HIUS is employed in various applications, including the extraction of bioactive compounds, inactivation of microorganisms and enzymes, and modification of proteins and carbohydrates. However, one of the primary challenges in HIUS processing is temperature control, which is essential for maintaining food stability, quality, and safety. Uncontrolled temperature increases can jeopardize these attributes. In this study, we assessed actual temperature conditions during HIUS treatments by analyzing thermal histories and investigating strategies for minimizing heat generation, such as pulsed ultrasound, ice baths, and combining sonication with external heating. We also evaluated the temperature profiles in fluids with varying thermophysical properties. While heat minimization techniques are effective in mitigating excessive heating, failure to account for thermal histories can lead to underestimations of the thermal effects. Accurate temperature monitoring provides critical insights into optimizing process design. Moreover, we observed potential solvent phase changes at the microscale during high-intensity treatments. These findings offer valuable guidance for improving heat management in HIUS applications and propose standardized methods for reporting thermal conditions and energy parameters in studies that utilize this technology.

## Graphical abstract

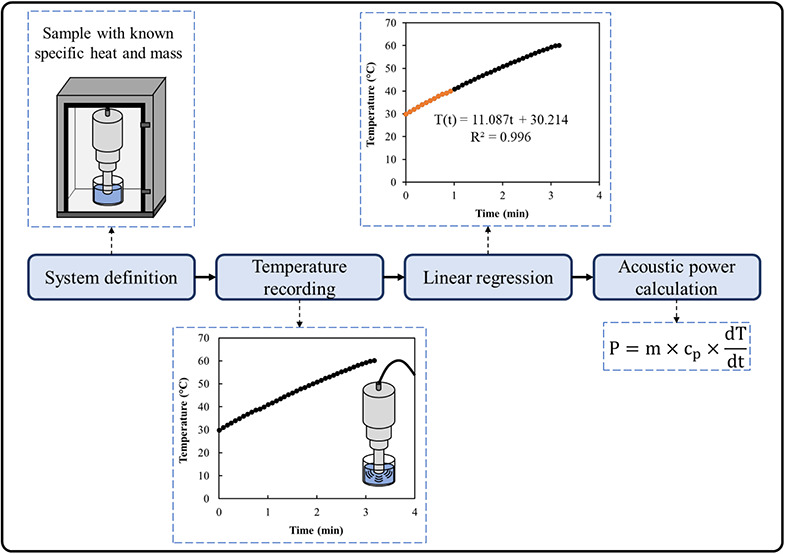

## 1 Introduction

High-intensity ultrasound (HIUS) has emerged as a promising technology in food engineering due to its ability to enhance extraction processes and inactivate microorganisms and endogenous enzymes without the use of excessive heat.^1–3^ HIUS operates through the generation of acoustic waves at frequencies typically between 20 and 100 kHz, which produce rapid compression and expansion cycles in the liquid medium they travel through. This creates microscopic cavitation bubbles that grow and collapse violently, releasing localized high temperatures and pressures.^4^ The mechanical effects of cavitation include shear forces, microstreaming, and shock waves, which can disrupt cell walls, enhance mass transfer, and facilitate the extraction of intracellular compounds. Additionally, the localized heat generated during bubble collapse can contribute to microbial and enzymatic inactivation, as well as the alteration of food structures, thereby contributing to the overall efficiency of HIUS in various food processing applications.^5^

Despite its potential, there is a significant gap in understanding the true thermal effects associated with HIUS treatments. This lack of clarity poses a challenge in optimizing these processes as the thermal history of food products undergoing HIUS is often inadequately reported. The precise control and monitoring of heat during HIUS treatments are critical steps, especially given the sensitivity of bioactive compounds and the need to maintain food stability and safety.^6^

Many studies have demonstrated the efficacy of HIUS in various food processing applications. However, the implicit assumption that HIUS operates under predominantly nonthermal mechanisms can lead to underestimating the thermal contributions and their impact on food quality. This oversight is particularly problematic in extraction processes, as well as microbial and enzymatic inactivation, where uncontrolled thermal effects could compromise the integrity of bioactive compounds and overall food safety.^2,7^ Consequently, a thorough thermodynamic analysis of HIUS processes is essential to fully understand and manage the heat generated during treatment.

In this context, the objective of this work is to provide a practical guide for determining the acoustic power delivered during HIUS processing, which is essential for the correct scaling and design of ultrasound-assisted processes. Additionally, this work aims to address the thermal effects associated with HIUS, challenging the common misconception that HIUS is a purely nonthermal technology. By assessing the temperature conditions and thermal histories in various HIUS treatments, the study offers valuable insights for process optimization, ensuring more accurate control over both acoustic energy delivery and heat management. Ultimately, the findings of this paper seek to improve the reporting and understanding of energy parameters in studies utilizing HIUS technology in food applications.

## 2 Methodology

### 2.1 Materials

In the experiments described below, HIUS treatments were conducted using different fluids, namely distilled water, 99.5% ethanol, sunflower oil, and clarified apple juice. The preparation of clarified apple juice (Loop Aromas e Sucos Concentrados, Piracicaba, Brazil) was carried out by diluting concentrated apple juice in distilled water at a ratio of 1:3 (17.7 °Brix). The experiments described in Sections 2.5–2.7 were conducted using only clarified apple juice.

### 2.2 Equipment

All experiments were carried out using the Ultrasonic Processor VCX 750 equipment (Sonics & Materials, Inc., USA), which consists of a power generator and a transducer coupled to a probe with a diameter of 13 mm, with a nominal power of 750 W and operating at 20 kHz. The equipment provides adjustable amplitude ranging from 20 to 100%. The temperature was monitored and recorded during the treatments by using a data logger Testo 175T3 (Testo SE & Co KGaA, Germany) attached to a thermocouple sensor, which was inserted into the sample during the sonication treatment. Figure 1 presents an illustration and specifications of the ultrasonic processor. In all treatments, sonication was applied inside an acoustic insulation chamber. Before each treatment, the probe was inserted into an ice bath for 30 s to standardize the initial condition of the probe.

**Figure 1:**
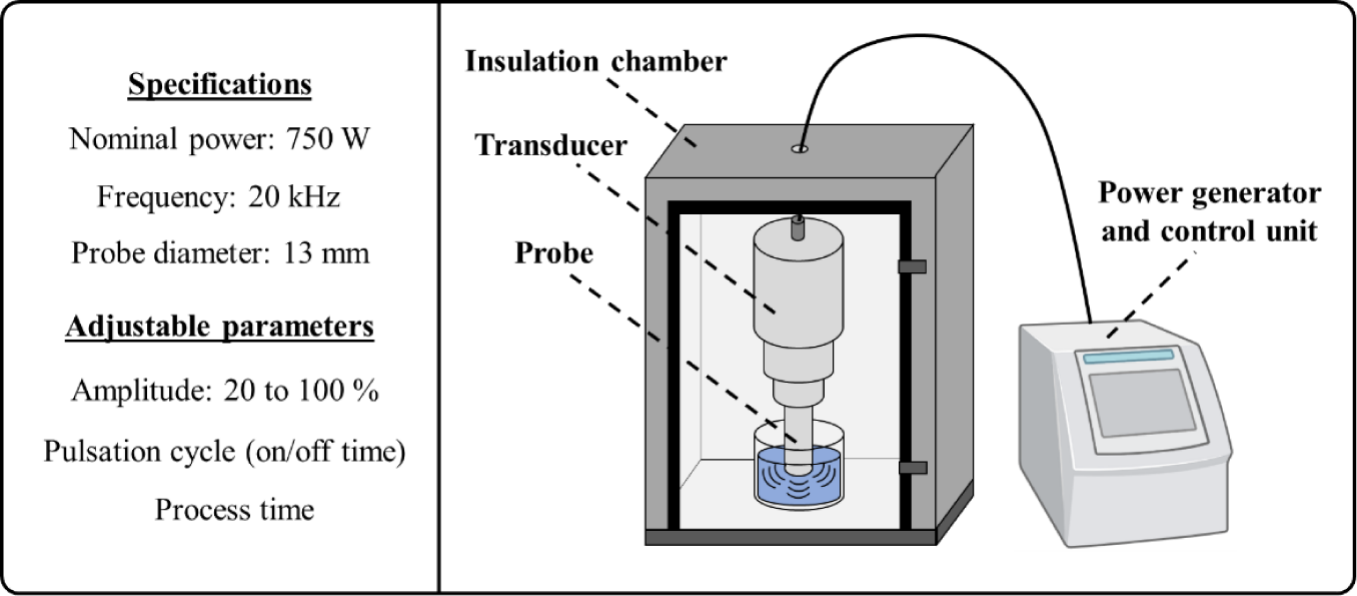
Schematic illustration of the ultrasonic processor system.

### 2.3 Calorimetric Assay to Determine the Acoustic Power and Intensity

The acoustic power (eq 1) or actual power provided by the HIUS system was determined for several amplitude values using a calorimetric assay according to the methodology described by Mason, Lorimer, Bates, and Zhao.^8^ For the calorimetric assay, a fixed and known mass of water was treated, and the temperature was recorded during the process. The calorimetric test was conducted according to the steps described below, and Figure 2 summarizes these steps.

**Figure 2:**
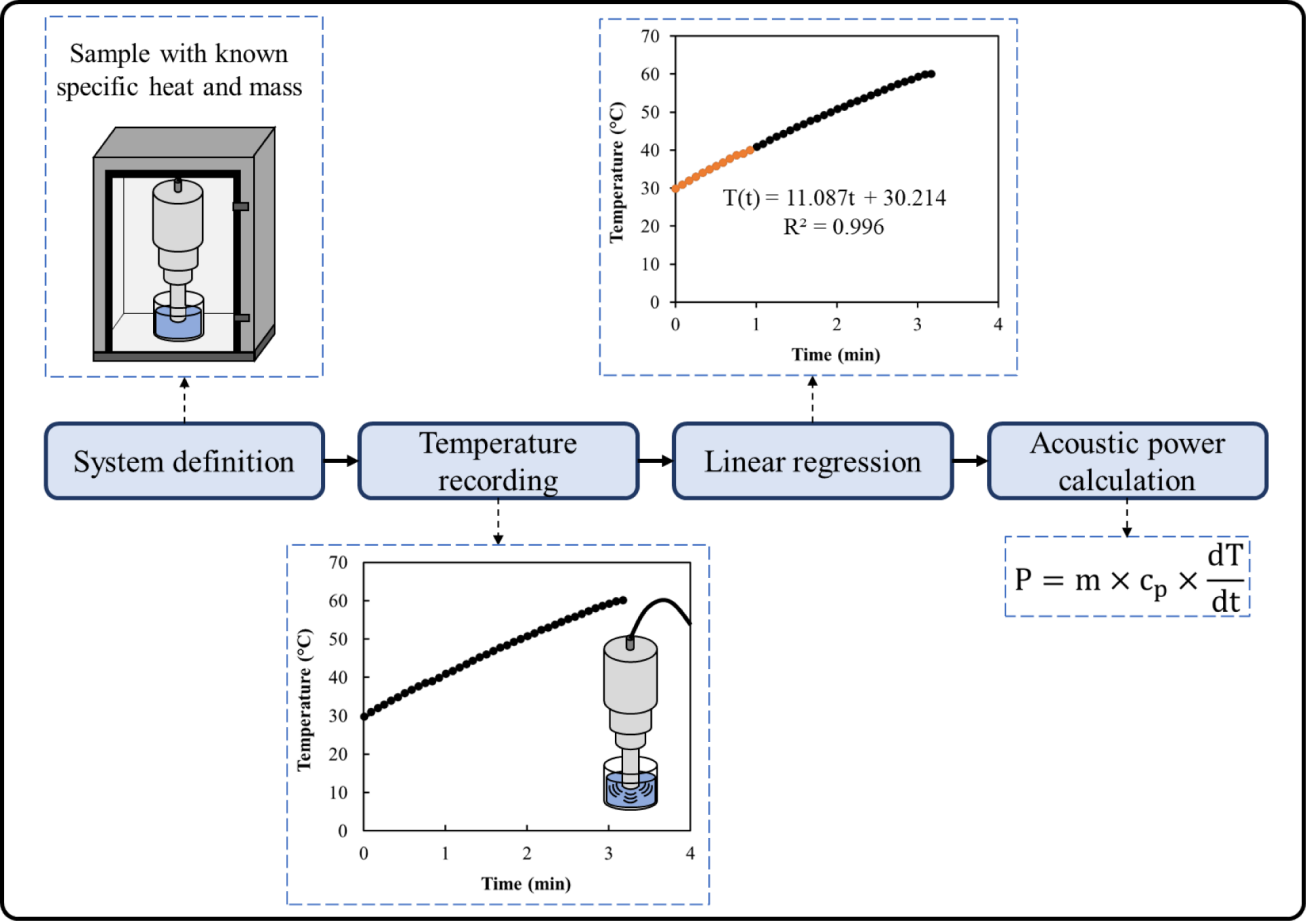
Steps of the calorimetric assay to determine the acoustic power in HIUS applications.

#### 2.3.1 System Definition

A sample with known thermophysical properties and mass was selected. In this study, 50 g of water was utilized as the sample. The specific heat capacity was considered constant at 4.18 J/g°C within the temperature range applied in this test. The calorimetric test for determining the acoustic power is based on the assumption that the system is adiabatic, meaning it does not exchange heat with its surroundings. The acoustic insulation chamber utilized in this study also helped reduce heat loss to the external environment. In addition to having a low thermal conductivity (approximately 0.03 W/mK), the air inside the chamber is also minimally moving, which results in very low heat transfer.

#### 2.3.2 Temperature Recording during HIUS Application

HIUS were applied at amplitudes of 20%, 30%, 40%, 50%, 60%, 70%, 75%, and 80% in duplicate (*n* = 2) until the samples reached 60 °C. The initial temperature of the samples was fixed at 30 °C before the treatments by using a thermostatic bath CORIO CD (Julabo, USA). During the treatments, the temperature was recorded every 5 s, and the temperature curves were plotted against time.

#### 2.3.3 Linear Regression to Determine the Rate of Temperature Change Over Time

To determine the rate of temperature change over time, the function T­(t) was derived from the linear portion of the temperature curves using linear regression. The linear period was observed experimentally during the initial minutes of the treatment. In this part of the curve, the rate of temperature increase is constant and can be obtained by the differential of T­(t), namely d*T*/d*t*. The linear regression was performed by fitting trend lines to data points within the 30–40 °C interval, ensuring at least 10 points from the linear period. The models obtained from the regression follow the general form, as described in eq 1, with a coefficient of determination (R²) of at least 0.99. The rate of temperature change over time was obtained through the angular coefficient of the linear models, according to eq 2

1 T(t)=at+b

2 $$\frac{\mathrm{dT}}{\mathrm{dt}}=a$$

where T is the temperature (°C), t is the time (s), a is the angular coefficient or the rate of change of temperature (°C/s), b is the linear coefficient or the temperature at time zero, and d*T*/d*t* is the rate of temperature change (°C/s).

#### 2.3.4 Acoustic Power Calculation

The acoustic power in W was calculated by eq 3

3 $$P=m\times C_{P}\times \frac{\mathrm{dT}}{\mathrm{dt}}$$

where P is the acoustic power (W), m is the mass of water (g), and C_P_ is the known specific heat capacity of water, equal to approximately 4.18 J/g°C.

The acoustic intensity was also determined according to eq 4 ^9^

4 $$\mathrm{AI}=\frac{4\times P}{\pi \times D^{2}}$$

where AI is the acoustic intensity (W/cm²) and D is the probe diameter (cm).

### 2.4 Thermal History of Different Fluids Treated by HIUS

To generate the thermal history for sonicated water, clarified apple juice, and ethanol, 100 g of each sample was treated for 15 min at 43.1 ± 0.2 W in duplicates. The sunflower oil was treated under the same conditions, but the treatment was interrupted when the temperature reached 90 °C to avoid overheating. The temperature was monitored and registered by the data logger at a 10 s frequency.

The specific energy was calculated using the acoustic power, processing time, and sample mass according to eq 5

5 $$S=\frac{P\times t}{m}$$

where S is the specific energy (J/g).

Linear regression was applied to the first 3.5 min of the thermal histories to estimate the rate of temperature change, d*T*/d*t* (in °C/min), as described in Section 2.3.3, for the four sonicated fluids evaluated. The model was applied to duplicates, and the rate was expressed as the average value and standard deviation.

### 2.5 Thermal History for Pulsed and Continuous HIUS Processing

Clarified apple juice was treated with pulsed and continuous HIUS treatments. An acoustic power of 43.1 ± 0.2 W was applied to 100 g of juice for 10 min in four different processing protocols with symmetric on:off cycles of 0:0, 3:3, 5:5, and 10:10 s, codified as C, P3, P5, and P10, respectively. The actual treatment time for pulsed treatments accounted only for the period during which sonication was active. The initial temperature was 30 °C, and the temperature was monitored and registered at a 10 s frequency. The specific energy was calculated according to eq 5.

### 2.6 Thermal History of HIUS Treatments Using Heat Minimizing Strategy

An ice bath was used as a heat-reducing technique. The recipient containing the clarified apple juice was inserted into the ice bath during treatment, and continuous sonication was applied at acoustic powers of 14.5 ± 0.1, 26.9 ± 0.3, 37 ± 1, and 48 ± 1 W for 15 min. Both treatments, with and without the ice bath, were conducted in duplicate. The temperature was monitored and recorded by the data logger every 10 seconds. The specific energy was calculated according to eq 5.

### 2.7 Thermosonication Treatment

For thermosonication treatment, a 500 mL jacketed Becker with internal circulation was used as a recipient for the water bath, which served as the external heat source. A 250 mL plastic beaker containing clarified apple juice was inserted into the bath. The bath was maintained at a set temperature with the aid of a thermostatic circulator tank, CORIO CD (Julabo, USA). Thermosonication was applied at a fixed acoustic power of 43.1 ± 0.2 W and three different bath temperatures: 40, 50, and 60 °C. The initial temperature was fixed at 30 °C for every sample, and the temperature increase from 30 °C to the bath temperature was also recorded. Sonication began when the sample reached the bath temperature and was maintained for 15 min.

### 2.8 Statistics and Data Analysis

The data were analyzed descriptively. The thermal history curves and the acoustic powers were calculated by using the mean values of process duplicates.

## 3 Results and Discussion

### 3.1 Determination of Acoustic Power and Intensity

Table 1 presents the results of the acoustic power obtained experimentally through calorimetric testing. As expected, increasing the amplitude also resulted in an increase in acoustic power. The amplitude, usually expressed as a percentage, corresponds to the maximum displacement of the transducer during vibration. In probe systems, amplitude limits the displacement of the probe tip. Higher amplitudes represent a larger fraction of the nominal power required from the electrical supply system. Additionally, higher amplitude values are associated with greater intensities of ultrasonic treatment and increased agitation of the medium.^10^ However, the amplitude alone does not provide sufficient information about the actual energy delivered to the sonicated medium.

**Table 1:** Acoustic Power and Acoustic Intensity Values (Mean ± Standard Deviation) Determined for Different Amplitudes by a Calorimetric Assay Using Water

Amplitude (%)	Acoustic power (W)	Acoustic intensity (W/cm²)
20	8.4 ± 0.3	6.3 ± 0.2
30	14.5 ± 0.1	10.9 ± 0.1
40	21.1 ± 0.2	15.9 ± 0.1
50	26.9 ± 0.3	20.3 ± 0.3
60	33.8 ± 0.1	25.5 ± 0.1
70	37 ± 1	27.7 ± 0.5
75	43.1 ± 0.2	32.5 ± 0.2
80	48 ± 1	36 ± 1

In general, a HIUS system consists of the following components: power source, piezoelectric transducer, amplifier, and probe or bath.^11,12^ The power source receives electrical energy from the supply and converts it into a signal at a specific frequency. The piezoelectric transducer then converts this electrical signal into mechanical vibrations.^13,14^ The generated vibration is amplified and transmitted to the sonic medium through acoustic waves. The acoustic power, determined by calorimetric testing, provides an estimate of the energy actually being received by the sonicated medium.^14^ Acoustic power is delivered to the sample after a series of energy conversions within the HIUS equipment. The process begins with electrical energy being converted into mechanical energy, which culminates in acoustic energy transmitted to the sample in the form of ultrasonic waves.^12^

Dissipations occur during these processes of energy conversion, resulting in acoustic powers that are significantly lower than the respective nominal power that the equipment requires from the energy source. For instance, configuring the 750 W equipment at 70% amplitude (525 W) resulted in an acoustic power of approximately 37 ± 1 W, which is less than 5% of the nominal power. Recent studies that applied HIUS to assist extraction processes have reported the following parameters: nominal power, amplitude, frequency, and processing or holding time.^15^ However, if knowing the actual energy delivered to the sample is relevant for a specific application, the acoustic power determination and reporting of acoustic power are recommended.

Acoustic intensity and specific energy are parameters derived from acoustic power. Specific energy provides information on the actual energy per unit mass delivered during treatment, and it is the recommended parameter for making energy-based comparisons between different novel technologies or proposing scale-up studies.^16^ The acoustic intensity, determined by eq 4, ranged from 7.4 to 42 W/cm² (Table 1). This parameter depends on the acoustic power and the emission area of the ultrasonic waves.^17^ In the case of probe systems, the emission area corresponds to the cross-sectional area of the probe. Although there is no exact threshold, ultrasonic processing is classified as high intensity when the acoustic intensity exceeds 1–10 W/cm².^10,13^ Recent studies have also reported the energetic parameters of HIUS through acoustic intensity or acoustic density.^18^

### 3.2 Thermodynamic Assessment Based on Thermal History for Different Fluids

The thermal histories of HIUS treatments using different fluid samples are presented in Figure 3. The curves for water, clarified apple juice, and ethanol showed similar behavior: an initial linear period followed by a decrease in the temperature variation until reaching a tendency toward a constant value, as shown in Figure 3a. The oil did not exhibit this behavior within the evaluated temperature range, showing only a linear increase.

**Figure 3:**
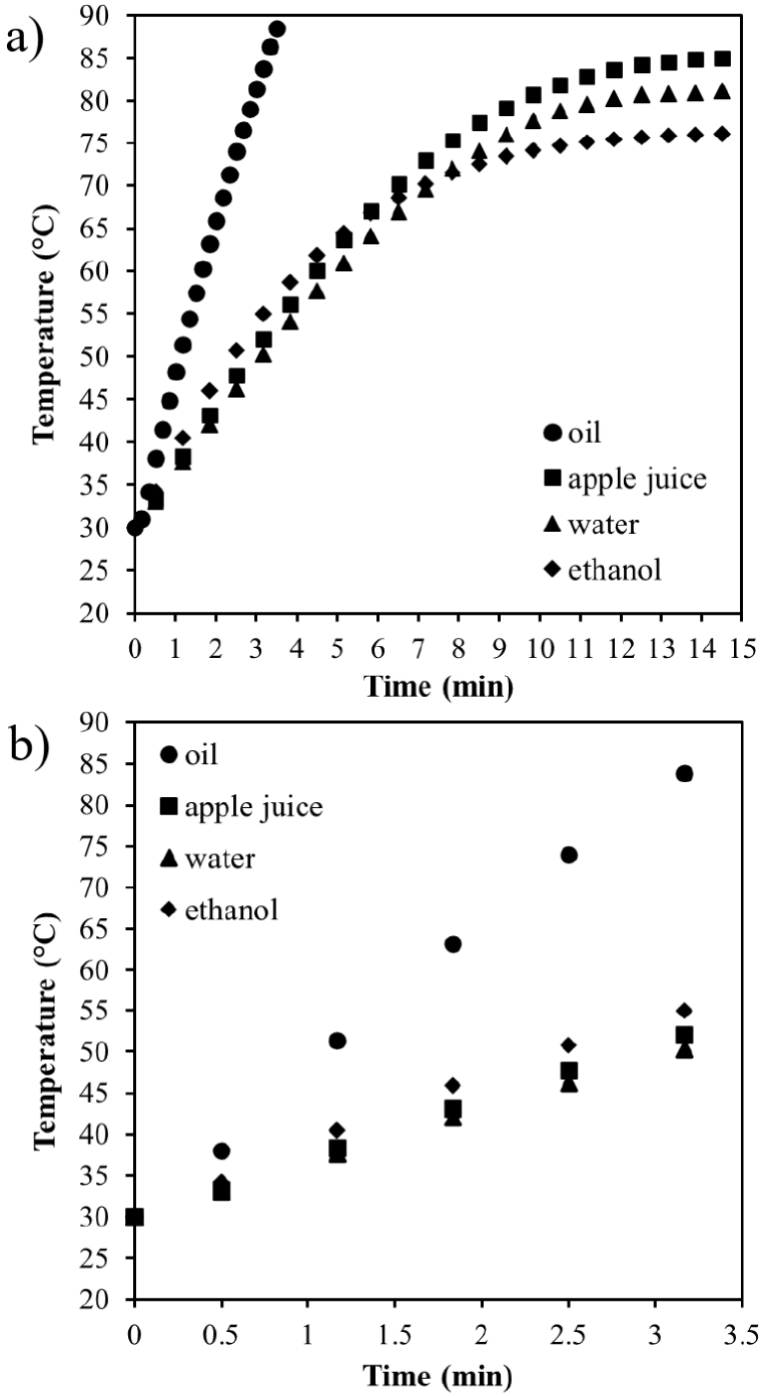
Thermal history of HIUS treatments at 43.1 ± 0.1 W applied to sunflower oil, clarified apple juice, water, and ethanol: (a) For 15 min and (b) during the first 3.5 min.

The nonlinearity of the curve, characterized by a falling temperature rate, becomes more pronounced approximately after 5, 8, and 9 min for ethanol, clarified apple juice, and water, respectively. At these times, the temperature reached approximately 65, 75,and 75 °C, respectively. The behavior of the curves indicates that if the treatments were sufficiently long, a horizontal line of constant temperature would be observed. This tendency of constant temperature is associated with a phase transition, from liquid to vapor, occurring inside the fluid systems. Although each fluid received the same specific energy of 259 J/g, they reached different temperatures after 15 min of sonication. This observation is expected, given that the fluids exhibit different thermal properties. Ethanol reached 76 °C after 15 min of sonication, which is close to its boiling point at atmospheric pressure, approximately 78 °C. The water sample showed 81 °C after 15 min of sonication, while clarified apple juice reached a higher temperature of 85 °C. The soluble solids in the juice enhance the boiling point, which might explain the higher temperature after 15 min of sonication. This effect is especially relevant because most applications involve samples with dissolved compounds, i.e., extraction, emulsion formation, and stabilization.

The temperature of the sunflower oil increased almost linearly and reached 90 °C in less than 4 min of sonication (Figure 3). As mentioned, the sonication was interrupted at this temperature to avoid overheating of the system and the recipient. If the process were kept for enough time, the oil would probably be heated to its smoke point of approximately 230 °C.^19^ The smoke point represents the temperature at which the oil starts to degrade and liberate smoke.^20^

The different slopes of the curves in the initial minutes of sonication result from the distinct properties of the fluids studied, as shown in Figure 3b. Oil showed a steeper slope compared to water, with rates of temperature change of 17.3 ± 1.0 and 6.5 ± 0.1, respectively. This difference can be attributed to the specific heat capacity of sunflower oil (2.43 J/g °C) being lower than that of water (4.18 J/g °C). Therefore, it is expected that oil heats faster than water. Ethanol, however, showed a lower rate compared to oil, although the specific heat of ethanol (2.46 J/g °C) is very close to that of oil. However, the heating rate of ethanol was 8.0 ± 0.2, which is closer to the rate observed for water despite their distinct specific heats.

It is possible that ethanol, the most volatile of the studied fluids, experienced phase change effects at a microscopic level during sonication, which did not occur for oil. This effect may have started in the first few minutes of HIUS treatment. Furthermore, although ethanol is expected to heat up more rapidly, it also undergoes vaporization effects more easily compared to water. Acoustic cavitation itself is known for causing extreme temperature conditions of around 5000 K (4726.85 °C) at a microscopic level.^10,21^ The transition from a constant rate to a falling temperature rate is a consequence of the phase transition that starts to occur microscopically. However, the sensor measures the mean temperature of the bulk medium, which might explain why this change in the curve behavior was observed before the boiling point of the fluids tested. The steady-state behavior observed in the thermal histories at 15 min of sonication, characterized by the tendency toward a constant temperature, may be associated with this phase transition occurring within these fluid systems.

These observations show that the linear period is limited by the thermophysical properties of the sample. During phase transition, the energy absorbed by the sample (latent heat) is not associated with a temperature change. In this situation, the information about the energy provided to the sample cannot be estimated by a calorimetric test, which impacts the acoustic power determination. As shown by the methodology used in this study, the calorimetric assay is based on the linear part of the thermal history. This becomes more critical at higher amplitudes, where the heating is faster. Therefore, temperature recording is a useful tool to understand the thermal behavior of a specific fluid sample during HIUS treatment.

### 3.3 Thermodynamic Assessment Based on Thermal History for Pulsed and Continuous Processing

The application of HIUS in pulses, involving on:off cycles, has been used primarily to mitigate thermal stress on the samples and reduce energy consumption.^22^ Figure 4 depicts the thermal profiles obtained following continuous HIUS treatment and the pulsed mode under three different conditions. The three pulse cycles evaluated produced similar curves. The processing time for all four treatments is 10 min, although the total time for pulsed treatments in symmetric on/off configurations is double that of continuous treatment. The sample temperatures after 10 min (20 min total time) reached values of 66.7, 66.5, and 67.7 °C for the 3:3-, 5:5-, and 10:10-s pulsed treatments, respectively. Continuous HIUS resulted in a temperature of 74.7 °C after 10 min.

**Figure 4:**
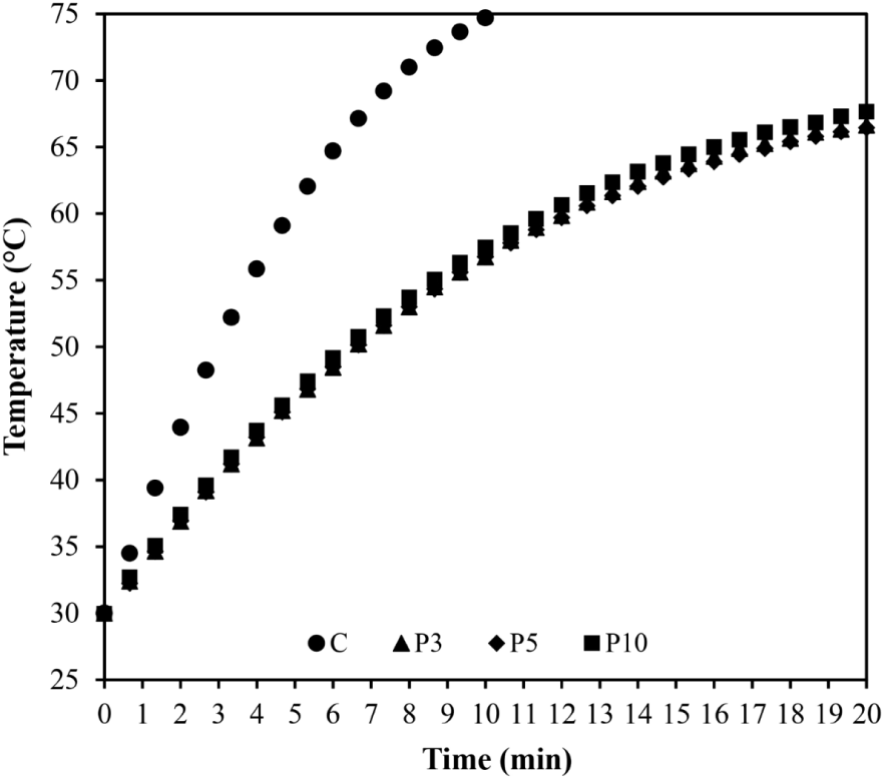
Thermal history of clarified apple juice treated by 43.1 ± 0.1 W using different pulsating protocols, where C is the continuous treatment and P3, P5, and P10 are treatments with 3:3-, 5:5-, and 10:10-s on:off protocols, respectively.

According to eq 5, the specific energy is the same for all treatments, approximately 259 J/g. However, pulsed sonication exhibited lower temperatures after applying this specific energy (10 min), demonstrating the functionality of the pulsed mode in preventing sample overheating during HIUS treatment. During the off stages, there is heat exchange between the system and the surroundings, causing the system to undergo brief cooling, which does not happen in continuous mode. Although the pulsing protocols had different on:off intervals (3, 5, and 10 s), the thermal behavior of the three curves remained similar. The temperature evolution during pulsed ultrasound was likely more influenced by the specific energy of the treatment, which was consistent across all protocols, rather than by the on:off intervals. One potential application of this operational feature is in the extraction of compounds sensitive to high temperatures, thereby preventing losses. Additionally, food quality can be better preserved in moderated process temperatures.^2^ Thermization of milk is applied as a gentle thermal treatment to reduce the pathogenic microbial load without significantly affecting the sensory and functional quality of the raw material. Generally, this process is conducted at 57–68 °C for 10–20 s.^23^ In this study, to establish a threshold above which the process is considered thermal, a temperature of 57 °C was selected.

According to the thermal histories in Figure 4, both the pulsed and continuous modes reached 57 °C in approximately 5 min of sonication. However, the total treatment time for the pulsed mode was twice as long (10 min). Therefore, a faster process with the same specific energy could be applied in continuous mode and result in the same temperature of 57 °C. This observation suggests that the effect of the pulsed mode in reducing heating may be more advantageous in longer processes, where higher temperatures can be reached in the continuous mode. Therefore, the importance of temperature registration for designing the HIUS process with pulsed mode should not be neglected. Recent studies have utilized the pulsed mode in ultrasound-assisted extractions. For example, Yeasmen and Orsat^24^ conducted protein extraction assisted by HIUS using a probe system in a 5:5 pulsed mode (on/off). The reported conditions included 120 kJ of energy, 60% amplitude, 25 °C temperature, and a 15-min treatment time. Additionally, the treatment was assisted by an ice bath, resulting in a recorded average temperature of 23 °C. Another study investigated the effect of pulsation in HIUS-assisted extraction of bioactives from pomegranate peel.^25^ A significant effect was observed, with the optimal extraction condition found at an 80% duty cycle (amplitude of pulse divided by pulse duration) and 6 min of treatment time.

### 3.4 Thermodynamic Assessment Based on Thermal History Using Ice Bath during Treatment

The thermal profiles of the sonicated samples with and without an ice bath are presented in Figure 5 for the acoustic powers studied. Compared with treatments without an ice bath, all samples processed with this heat reduction strategy showed lower temperatures. At 15 min, the temperatures were reduced by 43.3%, 34.1%, 27.3%, and 25.1% for acoustic powers of 14.5 ± 0.1, 26.9 ± 0.3, 37 ± 1, and 48 ± 1 W, respectively. Also, at 15 min, the samples subjected to acoustic powers of 14.5 ± 0.1, 26.9 ± 0.3, and 37 ± 1 W with an ice bath resulted in temperatures below the thermal threshold of 57 °C, reaching 31.4, 45.5, and 56.5 °C, respectively. The sample treated with 48 ± 1 W with an ice bath resulted in 59.9 °C in the 15-min process. Treatments without an ice bath reached the temperature of 57 °C in approximately 7.5, 5, and 4 min for acoustic powers of 26.9 ± 0.3, 37 ± 1, and 48 ± 1 W, respectively. Thus, the application of an ice bath allows for an increase in processing time while maintaining milder temperature conditions.

**Figure 5:**
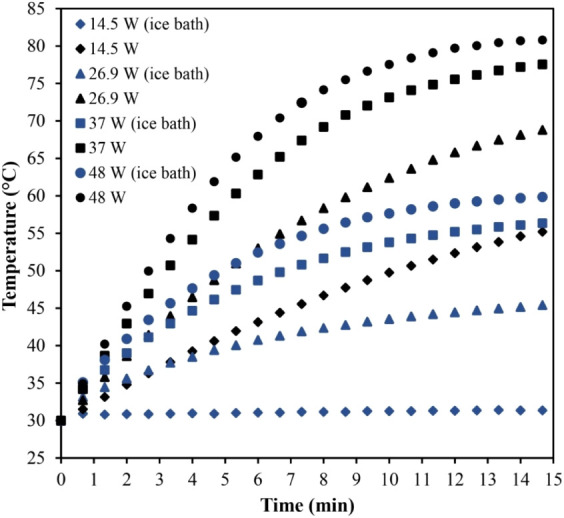
Thermal history of clarified apple juice treated by HIUS with and without an ice bath for acoustic powers ranging from 14.5 ± 0.1 to 48 ± 1 W.

The heating decrease due to the ice bath results from the intensified heat transfer between the system (sonicated medium) and the surrounding environment (ice bath) through the boundary (container wall). The greater temperature difference between the system and the ice bath leads to higher rates of heat transfer by conduction and convection, which removes more heat from the system. During the phase transition, the ice absorbs the latent heat of fusion at a constant temperature and pressure. Thus, as long as the phase transition occurs, the bath temperature tends to remain constant. Any temperature increase observed in the sonicated system results from the net rate of contribution of applied acoustic power and the rate of heat removal by the ice bath. As shown in Figure 5, the temperature profiles of the samples treated with an ice bath exhibited a lower slope in the linear period of the curve compared to the conventionally treated samples. Particularly at 14.5 ± 0.1 W, the temperature remained almost constant throughout the entire process, indicating that the energy transferred to the sample was removed roughly at the same rate.

Ice baths or circulation of cold water in a jacketed recipient are commonly used as techniques to avoid excessive heating during sonication, especially when the goal is to minimize degradation of heat-sensitive target compounds or simply to set the process temperature at a moderate value. Studies usually report that the temperature was kept constant by using an ice bath or circulating cold water during ultrasound treatment.^25,26^ However, without monitoring the process temperature through thermal histories, the actual temperature condition of the sample during treatment remains unknown, potentially leading to an underestimation of the heating.

### 3.5 Thermodynamic Assessment Based on Thermal History for Thermosonication Treatment

The thermosonication curves for the three temperatures evaluated are presented in Figure 6. The samples were preheated from 30 °C to the bath temperature. Therefore, the curves observed above the line corresponding to the bath temperatures result from the simultaneous effects of sonication at 43.1 ± 0.2 W and external heating. The samples reached temperatures of 71.7, 76.0, and 80.1 °C after 15 min of processing with bath temperatures of 40, 50, and 60 °C, respectively, which represent increases of 79.3%, 52.0%, and 33.5% compared to the bath temperatures.

**Figure 6:**
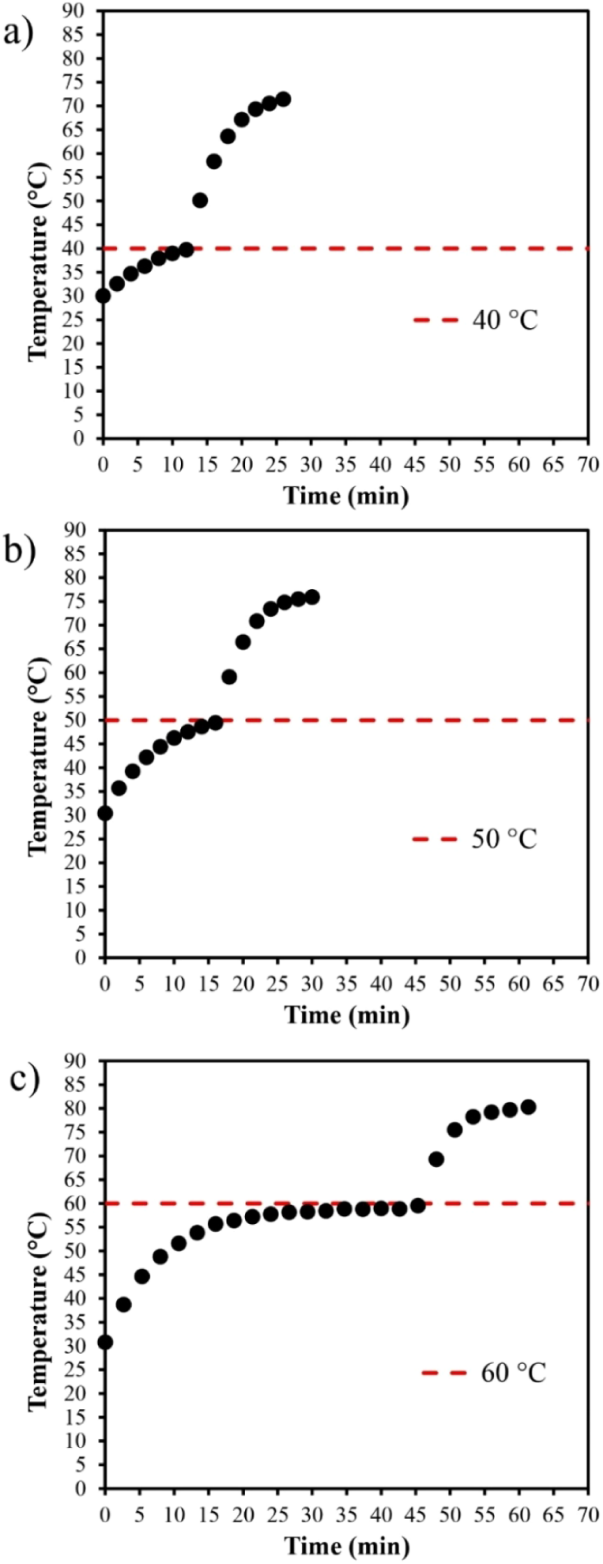
Thermal history of clarified apple juice treated by thermosonication applying an acoustic power of 43.1 ± 0.2 W: (a) heating bath at 40°C; (b) heating bath at 50°C; and (c) heating bath at 60°C.

Thermosonication involves the application of acoustic energy combined with moderate heating.^1^ This technique has been used for various purposes, such as the inactivation of microorganisms and enzymes, as well as assisting in the extraction of bioactive phytochemicals.^27,28^ In general, sonication at room temperature is not sufficient to inactivate most enzymes and microbial cells relevant in foods, and the combination with an external heat source is necessary.^29^ As shown in other results of this study, the thermal history plays an important role in determining the actual temperature in HIUS treatments and should be considered an essential tool in thermosonication applications. The thermal history information is particularly relevant for thermal treatments aimed at ensuring the stability and safety of food, such as the inactivation of microorganisms. It is also important to note that high temperatures can result in undesirable effects on the sensory and nutritional characteristics of food; therefore, temperature information can help optimize the treatments.

Positive results from thermosonication can also be achieved in the extraction of bioactive phytochemicals. Feihrmann et al.^28^ studied the extraction of phenolic compounds and anthocyanins from Tradescantia zebrina using HIUS with a probe system at temperatures of 40, 50, and 60 °C. Heating was conducted using a jacketed flask connected to a thermostatic bath. The optimal condition identified was 60 °C for 6.25 min with an amplitude of 20%. As discussed by the authors, the temperature positively impacted extraction by enhancing mass transfer phenomena. Temperature aids in reducing solvent viscosity, increasing the solubility of target compounds, and enhancing diffusion through the plant matrix. However, if the temperature reaches sufficiently high values, thermal degradation of the biomolecule being extracted may occur.^7^ The results obtained in this study show that the bath temperature is significantly exceeded, which can be detrimental for certain applications.

## 4 Conclusion

This study demonstrated that HIUS can induce significant temperature increases in treated media, challenging the notion that it is strictly a nonthermal process. Our findings highlight that if sonication is applied for a sufficiently long duration, phase transitions can occur at the microscale, altering the thermal behavior of the system. This has critical implications for process optimization, particularly in applications where excessive heating may compromise the stability and functionality of bioactive compounds.

The evaluation of different processing strategies, including pulsed ultrasound and ice bath cooling, confirmed their effectiveness in mitigating excessive heating. However, the thermal history curves revealed that the efficiency of these approaches depends on both the specific energy applied and the thermophysical properties of the fluid. Therefore, temperature monitoring should be considered an essential tool for designing HIUS processes, especially in food applications, where thermal control is crucial for quality preservation.

Furthermore, this study provides a detailed protocol for estimating the actual power delivered to the system and calculating specific energy, which are key parameters for energy-based comparisons between novel technologies and scale-up studies. Standardizing the reporting of these parameters will contribute to more accurate assessments of HIUS effects in both research and industrial applications.

Future studies should explore the chemical effects of HIUS, such as the generation of reactive species and their impact on food stability. Additionally, investigating HIUS-induced phase transitions at a molecular level could provide further insights into their thermodynamic effects. By improving the understanding and reporting of thermal conditions in HIUS treatments, this work aims to support the development of more precise and efficient applications of ultrasound technology in food processing and beyond.
